# One-pot and metal-free synthesis of 3-arylated-4-nitrophenols via polyfunctionalized cyclohexanones from β-nitrostyrenes

**DOI:** 10.3762/bjoc.16.150

**Published:** 2020-07-22

**Authors:** Haruyasu Asahara, Minami Hiraishi, Nagatoshi Nishiwaki

**Affiliations:** 1 School of Environmental Science and Engineering, Kochi University of Technology, Tosayamada, Kami, Kochi 782-8502, Japan; 2 Research Center for Molecular Design, Kochi University of Technology, Tosayamada, Kami, Kochi 782-8502, Japan; 3 Graduate School of Pharmaceutical Sciences, Osaka University, Yamadaoka 1-6, Suita, Osaka 565-0871, Japan

**Keywords:** 3-arylated-4-nitrophenol, Danishefsky’s diene, Diels–Alder reaction, nitroalkene, polysubstituted cyclohexanone

## Abstract

β-Nitrostyrenes underwent a Diels–Alder reaction with Danishefsky’s diene to afford cyclohexenes together with the corresponding hydrolyzed products, 3-arylated-5-methoxy-4-nitrocyclohexanones. When the reaction was conducted in the presence of water, the cyclohexenes were efficiently hydrolyzed into cyclohexanones. Subsequent aromatization by heating the cyclohexanone with a catalytic amount of iodine in dimethyl sulfoxide gave 3-arylated-4-nitrophenols. The reaction of nitrostyrenes with Danishefsky’s diene could be conducted in one pot to directly afford the corresponding nitrophenols. Moreover, a heteroaryl group, e.g., a thienyl group could be introduced into the nitrophenol framework.

## Introduction

The 4-nitrophenol framework is characterized by a biased electron density in the ring and an acidic hydroxy group, which can be attributed to the electron-donating hydroxy and the electron-withdrawing nitro groups. The derivatives of 4-nitrophenol are widely used in various applications. In particular, 3-arylated-4-nitrophenols have attracted much attention from a biological viewpoint [1–11]; however, they cannot be synthesized by the direct modification of 4-nitrophenol because the *ortho*-directing hydroxy and the *meta*-directing nitro group hinder the electrophilic modification at the 3-position. Generally, the aryl group is introduced by a Suzuki–Miyaura cross-coupling reaction [4–5], for which 3-bromo-4-nitrophenol must be prepared by the nitration of 3-bromophenol [6–7]. An alternative approach is the nitration of 3-arylphenol [8–9]. However, these nitration methods are less effective because the yield of the desired product is reduced by the formation of regioisomers. Although the hydroxylation of 3-arylated-1-fluoro-4-nitrobenzene has also been reported as a related strategy, multistep reactions are necessary for preparing the precursor [10]. The condensation of benzyl methyl ketone with a nitrovinamidinium salt also affords 3-arylated-4-nitrophenols; however, 3-arylated-*N*,*N*-dimethyl-4-nitroanilines are competitively formed in this reaction [11]. Hence, there is an urgent demand for the development of a facile method toward the preparation of 3-arylated-4-nitrophenols.

**Scheme 1:**
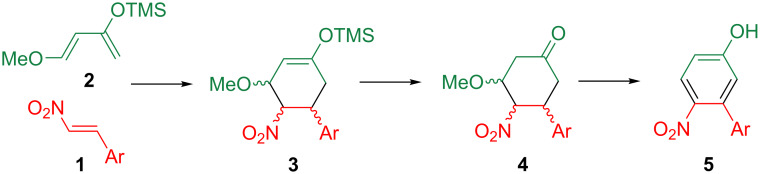
Synthetic scheme of the 3-arylated-4-nitrophenols **5**.

On the other hand, nitroalkenes possess an electron-deficient double bond, and hence, they serve as an excellent Michael acceptor and dienophile [12–19]. When β-nitrostyrene (**1**, Ar = Ph) is subjected to the Diels–Alder reaction, a C2 unit possessing a nitro group and an aryl group at the vicinal position is incorporated into the products. This unique reactivity prompted us to probe the synthesis of the 3-arylated-4-nitrophenols **5** by the Diels–Alder reaction of nitrostyrenes **1** with Danishefsky’s diene (**2**, the trimethylsiloxy group of the diene can be converted to a phenolic hydroxy group by hydrolysis) [20], followed by the oxidation and aromatization of the obtained cyclohexanone **4** (Scheme 1).

## Results and Discussion

**Figure 1:**
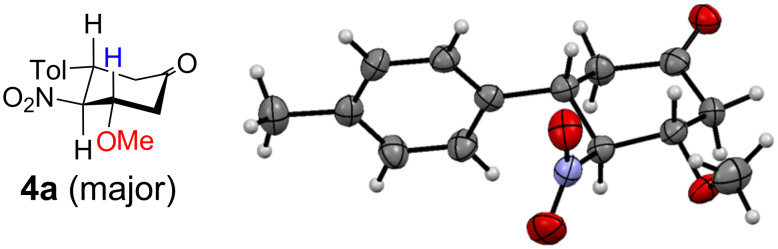
X-ray crystallography of the major isomer of **4a**. The thermal ellipsoids indicate 50% probability.

Heating an acetonitrile solution of nitrostyrene **1a** with Danishefsky’s diene (**2**) at 60 °C for 18 h afforded trace amounts of **3a** and **4a**, both of which were obtained as a mixture of diastereomeric isomers (Table 1, entry 1). The stereochemistry of the major product **4a** was determined to be *trans*,*trans* (all-equatorial) by X-ray crystallography (Figure 1). Among the solvents tested, less polar solvents, such as hexane and toluene, were found to be suitable for the reaction (Table 1, entries 1–5). Consequently, the total yield of **3a** and **4a** increased up to 82% when the reaction was conducted under reflux in toluene (Table 1, entries 5–7). The diastereomeric ratio increased when the reaction was conducted at temperatures higher than 90 °C, presumably due to the easier formation of the thermodynamically stable isomer. As described later, DMSO was found to be an effective solvent for the subsequent oxidation. However, toluene was determined to be the best solvent because the recovery of **1a** was observed even at 120 °C (Table 1, entry 8).

**Table 1:** Optimization of the reaction conditions for the Diels–Alder reaction.^a^

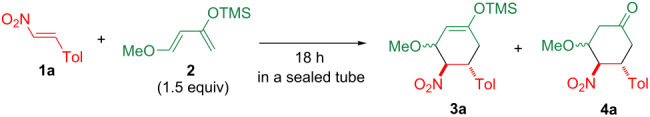						

entry	solvent	*T* [°C]	total yield [%]^b^	yield [%]^b^		dr^b,c^ of **4a**
					
				**3a**	**4a**	

1	MeCN	60	6	3	3	67:33
2	Et_2_O	60	21	20	1	50:50
3	CHCl_3_	60	41	31	10	50:50
4	hexane	60	39	28	11	55:45
5	PhMe	60	51	38	13	54:46
6	PhMe	90	61	45	16	83:17
7	PhMe	120	82	59	23	83:17
8	DMSO	120	30	30	0	–

^a^Tol: 4-MeC_6_H_4_. ^b^Determined by ^1^H NMR spectroscopy. ^c^Diastereomeric ratio.

**Scheme 2:**
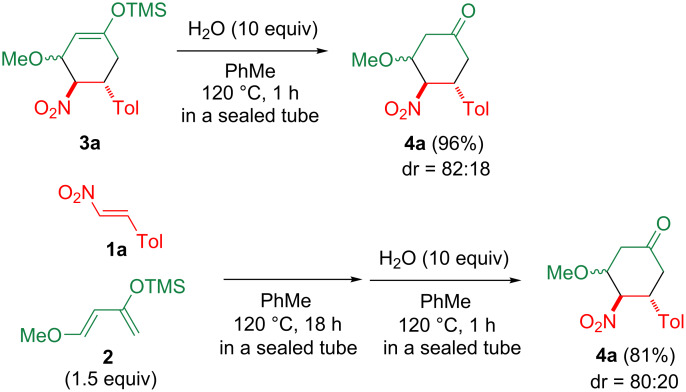
Conversion from **3a** to **4a** and one-pot synthesis of **4a**.

Cyclohexene **3a** was efficiently converted to the cyclohexanone **4a** upon heating at 120 °C in toluene in the presence of 10 equiv water (Scheme 2). This result prompted us to synthesize **4a** in one pot from **1a** and **2** by Diels–Alder reaction and subsequent heating with water (Scheme 2).

**Scheme 3:**
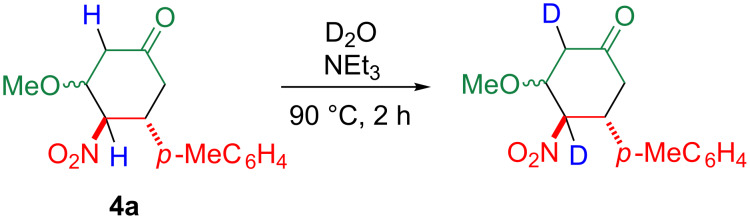
Deuteration of cyclohexanone **4a**.

Cyclohexanone **4a** has acidic hydrogen atoms that can facilitate the aromatization by modification, e.g., by iodination. In order to obtain further insights into this possibility, **4a** was heated with deuterium oxide, but no change was observed. In contrast, the signals assigned to the protons in the 4- and 6-position disappeared in the NMR spectrum when the mixture was heated in the presence of triethylamine, indicating that the α-protons of the carbonyl and nitro groups are acidic and easily modifiable (Scheme 3, see the NMR charts in Supporting Information File 1).

The aromatization of **4a** using iodine was then attempted (Table 2). The reaction did not proceed in toluene or acetonitrile (Table 2, entries 1 and 2), but dimethyl sulfoxide (DMSO) was effective for the aromatization, and nitrophenol **5a** was obtained in 26% yield (Table 2, entry 3) [21–23]. This reaction proceeded efficiently to afford **5a** in 61% yield even when the amount of iodine was decreased to 10 mol %. However further decreasing the iodine amount to 5 mol % was not effective for the conversion (Table 2, entries 4 and 5). In each case, both stereoisomers of **4a** were completely consumed, and several unidentified products were obtained.

**Table 2:** Aromatization of cyclohexanone **4a**.

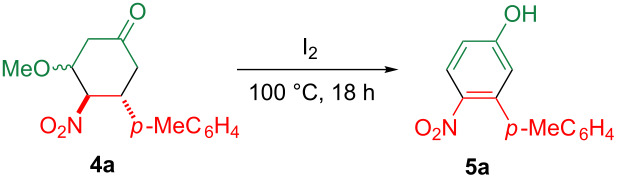			

entry	solvent	I_2_ [equiv]	yield [%]

1	PhMe	0.2	0
2	MeCN	0.2	0
3	DMSO	0.2	26
4	DMSO	0.1	61
5	DMSO	0.05	41

**Scheme 4:**
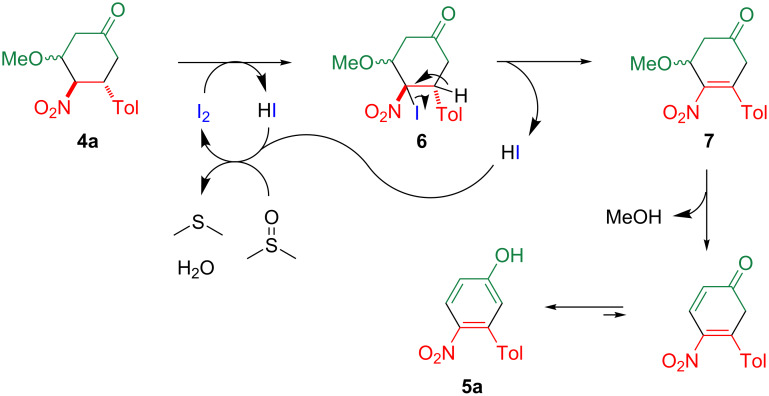
A plausible mechanism for the formation of **5a**.

The aromatization is considered to proceed as shown in Scheme 4. After the iodization at the 4-position, which leads to the formation of the intermediate **6**, the aromatization is achieved by the successive elimination of hydrogen iodide and methanol, with concurrent tautomerism to afford **5a**. The formed hydrogen iodide is easily oxidized by DMSO to regenerate iodine, so that the reaction can be performed with a catalytic amount of iodine.

**Figure 2:**
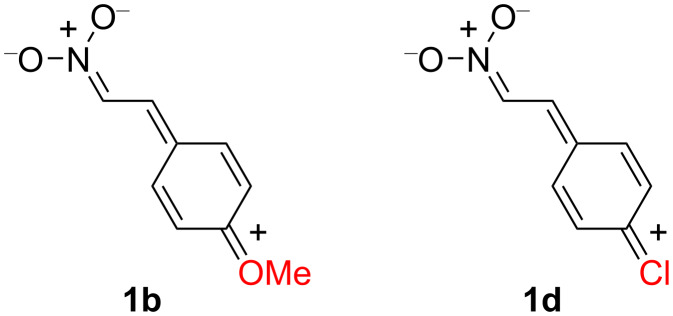
Resonance structure of nitroalkenes **1b** and **1d**.

The optimal conditions were applied to the one-pot three-step reaction of the other nitrostyrenes **1b**–**e**, and the corresponding 3-phenylated-4-nitrophenols **5b**–**e** were furnished in moderate yield (Table 3, entries 1–4). Among the nitrostyrenes employed, **1b** and **1d** had a lower reactivity, which was presumably due to the electron-donating resonance effect of the substituents. In these cases, the resonance contributor shown in Figure 2 diminished the nitroalkene properties and consequently suppressed the Diels–Alder reaction with **2**. It is noteworthy that not only the benzene ring, but also a heteroaromatic ring could be introduced into the nitrophenol framework by using this method (Table 3, entry 5).

**Table 3:** One-pot synthesis of 3-arylated-4-nitrophenols **5**.

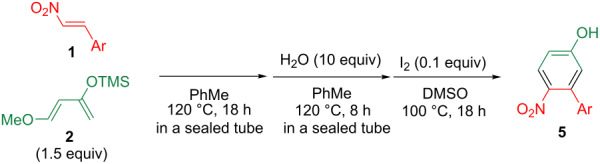			

entry	Ar	**2**/**5**	yield [%]

1	*p*-MeOC_6_H_4_	**b**	34
2	C_6_H_5_	**c**	69
3	*p*-ClC_6_H_4_	**d**	25
4	*p*-CF_3_C_6_H_4_	**e**	44
5	2-thienyl	**f**	39

## Conclusion

β-Nitrostyrene **1a** underwent a Diels–Alder reaction with Danishefsky’s diene (**2**) to afford polysubstituted cyclohexene **3a** and cyclohexanone **4a**. The addition of water to the reaction mixture accelerated the conversion from **3a** to **4a**. The oxidative aromatization of **4a** was achieved by the treatment with a catalytic amount of iodine in DMSO to furnish nitrophenol **5a**. This protocol was also applicable to other nitroalkenes, **1b**–**f**, to afford the corresponding 3-arylated-4-nitrophenols **5b**–**f**. This reaction could be conducted in one pot without using any transition-metal reagent. Since the starting nitroalkenes were prepared by the condensation of a (hetero)aryl aldehyde and nitromethane, an easy modification of the aryl group at the 3-position of the 4-nitrophenol is possible. Thus, the proposed reaction would be a useful tool for the elaborate synthesis of aromatic compounds.

## Experimental

### General

All reagents were purchased from commercial sources and used without further purification. ^1^H and ^13^C NMR spectra were recorded on a Bruker DPX-400 spectrometer (at 400 MHz and 100 MHz, respectively) or on a JEOL JNM-LA 500 spectrometer (at 500 MHz and 125 MHz, respectively) in CDCl_3_ using TMS as an internal standard. The assignments of the ^13^C NMR signals were performed by DEPT experiments. A Shimadzu IR spectrometer equipped with an ATR detector was used to record infrared spectra. High-resolution mass spectra were obtained on an AB SCIEX Triplet TOF 4600 mass spectrometer. Melting points were recorded on an SRS-Optimelt automated melting point system and aree uncorrected.

### Preparation of (*E*)-1-(4-methylphenyl)-2-nitroethene (**1a**)

To a solution of ammonium acetate (2.63 g, 34 mmol) in acetic acid (20 mL) were added nitromethane (5.25 mL, 98 mmol) and 4-methylbenzaldehyde (1.96 mL, 16 mmol), and the resulting mixture was heated at 100 °C for 6 h. After the addition of water (100 mL), the pH value was adjusted to 7 with a 2 M aqueous sodium hydroxide solution. This was extracted with ethyl acetate (50 mL × 3), and the organic layer was washed with brine (100 mL × 1), dried over magnesium sulfate, and concentrated to afford crude product (2.66 g) as yellow solid. The solid was recrystalized with a mixed solvent (hexane/dichloromethane 10:1) to afford nitrostyrene **1a** [24] (1.99 g, 12.2 mmol, 76%) as yellow needles. The other nitroalkenes **1b**–**f** [25–29] were prepared in the same way.

### One-pot Diels–Alder reaction of nitrostyrene **1a** and Danishefsky’s diene (**2**)

To a solution of Danishefsky’s diene (**2**, 129.2 mg, 0.75 mmol) in toluene (1 mL), nitrostyrene **1a** (81.6 mg, 0.50 mmol) was added, and the resultant mixture was heated at 120 °C for 18 h in a sealed tube. Water (90 mg, 5.0 mmol) was added, and the mixture was heated at 120 °C for further 1 h in a sealed tube. After the removal of the solvent under reduced pressure, the residue was treated by column chromatography using silica gel (hexane/ethyl acetate 9:1) to afford the major isomer of cyclohexanone **4a** (205 mg, 0.32 mmol, 78%) as yellow solid and the minor isomer of cyclohexanone **4a** (26.3 mg, 0.10 mmol, 10%, as a mixture with the major isomer, dr = 6:1) as pale yellow solid; however, further purification of the minor isomer could not be achieved.

Major isomer: pale yellow needles; mp 122–123 °C; ^1^H NMR (400 MHz, CDCl_3_, δ) 2.32 (s, 3H), 2.56 (ddd, *J* = 0.6, 11.1, 14.4 Hz, 1H), 2.63 (ddd, *J* = 2.0, 5.1, 15.2 Hz, 1H), 2.70 (ddd, *J* = 0.6, 13.4, 15.2 Hz, 1H), 3.07 (ddd, *J* = 2.0, 5.2, 14.4 Hz, 1H), 3.37 (s, 3H), 3.41 (ddd, *J* = 5.1, 11.6, 13.4 Hz, 1H), 4.09 (ddd, *J* = 5.2, 9.2, 11.1 Hz, 1H), 4.95 (dd, *J* = 9.2, 11.6 Hz, 1H), 7.08 (d, *J* = 8.0 Hz, 2H), 7.15 (d, *J* = 8.0 Hz, 2H); ^13^C NMR (100 MHz, CDCl_3_, δ) 21.0 (CH_3_), 42.8 (CH), 44.3 (CH_2_), 46.0 (CH_2_), 57.6 (CH_3_), 78.6 (CH), 93.6 (CH), 126.8 (CH), 130.0 (CH), 133.6 (C), 138.4 (C), 203.1 (C); IR (ATR) v_max_: 536, 821, 1091, 1340, 1550, 1718 cm^−1^; HRESIMS-TOF (*m*/*z*): [M + Na]^+^ calcd for C_14_H_17_NO_4_Na, 286.1050; found, 286.1040.

Minor isomer: ^1^H NMR (400 MHz, CDCl_3_, δ) 2.31 (s, 3H), 2.51 (ddd, *J* = 0.5, 12.4, 15.2 Hz, 1H), 2.69 (ddd, *J* = 0.5, 3.6, 15.2 Hz, 1H), 2.71 (ddd, *J* = 2.4, 5.6, 15.2 Hz, 1H), 2.95 (ddd, *J* = 2.4, 3.6, 15.2 Hz, 1H), 3.37 (s, 3H), 4.10 (ddd, *J* = 5.6, 12.4, 11.4 Hz, 1H), 4.43 (ddd, *J* = 3.0, 3.6, 3.6 Hz, 1H), 4.66 (dd, *J* = 2.8, 11.4 Hz, 1H), 7.14–7.16 (m, 4H); ^13^C NMR (100 MHz, CDCl_3_, δ) 21.0 (CH_3_), 39.9 (CH), 42.9 (CH_2_), 46.5 (CH_2_), 57.6 (CH_3_), 78.8 (CH), 89.0 (CH), 126.8 (CH), 129.8 (CH), 136.4 (C), 137.6 (C), 204.2 (C).

### Aromatization of cyclohexanone **4a**

To a solution of the cyclohexanone **4a** (52.6 mg, 0.2 mmol) in DMSO (1 mL), iodine (5.0 mg, 0.02 mmol) was added, and the resulting mixture was heated at 100 °C for 18 h. To the reaction mixture, a saturated aqueous solution of sodium thiosulfate (3 mL) was added, and the mixture was extracted with ethyl acetate (3 mL × 3). The organic layer was washed with brine (3 mL × 1), dried over magnesium sulfate, and concentrated to afford the crude product (39.4 mg) as brown oil. Further purification was performed with column chromatography on silica gel to afford 3-(4-methylphenyl)-4-nitrophenol (**5a**) [30], which eluted with hexane/ethyl acetate 8:2 (*R*_f_ 0.44, 28.0 mg, 0.12 mmol, 61%) as brown solid. ^1^H NMR (400 MHz, CDCl_3_, δ) 2.40 (s, 3H), 5.4–5.2 (br, 1H), 6.80 (d, *J* = 2.6 Hz, 1H), 6.85 (dd, *J* = 2.6, 8.8 Hz, 1H), 7.18 (d, *J* = 8.2 Hz, 2H), 7.22 (d, *J* = 8.2 Hz, 2H), 7.89 (d, *J* = 8.8 Hz, 1H); ^13^C NMR (100 MHz, CDCl_3_, δ) 21.2 (CH_3_), 114.4 (CH), 118.5 (CH), 127.2 (CH), 127.6 (CH), 129.3 (CH), 134.8 (C), 138.1 (C), 139.8 (C), 142.3 (C), 158.9 (C).

### One-pot synthesis of 3-arylated-4-nitrophenol **5b**

To a solution of Danishefsky’s diene (**2**, 129.2 mg, 0.75 mmol) in toluene (1 mL), nitrostyrene **1b** (90.0 mg, 0.50 mmol) was added, and the resulting mixture was heated at 120 °C for 18 h in a sealed tube. Water (90 mg, 5.0 mmol) was added, and the mixture was heated at 120 °C for further 1 h in a sealed tube. After the removal of the solvent under reduced pressure, the residue was dissolved in DMSO (2.5 mL), and iodine (12.7 mg, 0.05 mmol) was added. After heating at 100 °C for 18 h, a saturated aqueous solution of sodium thiosulfate (8 mL) was added, and the mixture was extracted with ethyl acetate (8 mL × 3). The organic layer was washed with brine (8 mL × 1), dried over magnesium sulfate, and concentrated. The residue was treated by column chromatography on silica gel to afford nitrophenol **5b**, which eluted with hexane/ethyl acetate 8:2 (41.7 mg, 0.17 mmol, 34%) as yellow solid. When the other nitroalkenes **1c**–**f** were used, the reaction was conducted in the same way.

## Supporting Information

File 1 Spectral data for **5b**–**f**, NMR spectra (^1^H, ^13^C, and DEPT) for **4a** and **5a**–**f**, and crystallographic data for **4a**.
