# Supplementary material for: One-pot and metal-free synthesis of 3-arylated-4-nitrophenols via polyfunctionalized cyclohexanones from β-nitrostyrenes
Source: Beilstein J Org Chem. 2020 Jul 22;16:1830–6. doi: 10.3762/bjoc.16.150 (PMC7385393; doi:10.3762/bjoc.16.150)
Supplement: File 1 — Spectral data for 5b–f, NMR spectra (1H, 13C, and DEPT) for 4a and 5a–f, and crystallographic data for 4a. [file Beilstein_J_Org_Chem-16-1830-s001.pdf]

BEILSTEIN JOURNAL OF ORGANIC CHEMISTRY

Supporting Information

for

One-pot and metal-free synthesis of 3-arylated-4-nitrophenols via polyfunctionalized cyclohexanones from β-nitrostyrenes

Haruyasu Asahara, Minami Hiraishi and Nagatoshi Nishiwaki

Beilstein J. Org. Chem. **2020,** *16,* 1830–1836. doi:10.3762/bjoc.16.150

Spectral data for 5b–f, NMR spectra (^{1}H, ^{13}C, and DEPT) for 4a and 5a–f, and crystallographic data for 4a

License and Terms: This is a supporting information file under the terms of the Creative Commons Attribution License (http://creativecommons.org/licenses/by/4.0). Please note that the reuse, redistribution and reproduction in particular requires that the authors and source are credited.
The license is subject to the *Beilstein Journal of Organic Chemistry* terms and conditions: (https://www.beilstein-journals.org/bjoc)

Table of Contents

Spectral data of **5b**–**f**		S2
X-ray analysis		S4
3-Methoxy-5-(4-methylphenyl)-4-nitrocyclohexanone (**4a**)	^{1}H, ^{13}C, DEPT NMR and deuteration experiment	S6
3-(4-Methylphenyl)-4-nitrophenol (**5a**)	^{1}H, ^{13}C, DEPT NMR	S8
3-(4-Methoxyphenyl)-4-nitrophenol (**5b**)	^{1}H, ^{13}C, DEPT NMR	S10
4-Nitro-3-phenylphenol (**5c**)	^{1}H, ^{13}C NMR	S12
3-(4-Chlorophenyl)-4-nitrophenol (**5d**)	^{1}H, ^{13}C, DEPT NMR	S13
3-(4-Trifluoromethylphenyl)-4-nitrophenol (**5e**)	^{1}H, ^{13}C NMR	S15
4-Nitro-3-(2-thienyl)phenol (**5f**)	^{1}H, ^{13}C, DEPT NMR	S16

Spectral data for **5b**–**f**

*3-(4-Methoxyphenyl)-4-nitrophenol (**5b**)*

Yellow solid, mp 137–138 °C. ^{1}H NMR (400 MHz, $CDCl_3$) δ 3.84 (s, 3H), 5.6–5.8 (br, 1H), 6.78 (d, *J* = 2.4 Hz, 1H), 6.85 (dd, *J* = 2.6, 8.8 Hz, 1H), 7.18 (d, *J* = 8.2 Hz, 2H), 7.22 (d, *J* = 8.2 Hz, 2H), 7.89 (d, *J* = 8.8 Hz, 1H); ^{13}C NMR (100 MHz, $CDCl_3$) δ 21.2 (CH_3), 114.4 (CH), 118.5 (CH), 127.2 (CH), 127.6 (CH), 129.3 (CH), 134.8 (C), 138.1 (C), 139.8 (C), 142.3 (C), 158.9 (C); IR (ATR/cm^{-1}) 530, 616, 828, 1316, 1506, 3343; HRMS (ESI-TOF) calcd. for (M^++Na^+) $C_{13}H_{11}NO_4Na$: 268.0580, found: 268.0593.

*4-Nitro-3-phenyl-phenol (**5c**)* [1]

Brown oil. ^{1}H NMR (400 MHz, $CDCl_3$) δ 5.88–5.98 (br, 1H), 6.80 (d, *J* = 2.8 Hz, 1H), 6.86 (dd, *J* = 2.8, 8.8 Hz, 1H), 7.24–7.30 (m, 2H), 7.38–7.43 (m, 3H), 7.93 (d, *J* = 8.8 Hz, 1H); ^{13}C NMR (125.5MHz, $CDCl_3$) δ 114.6 (CH), 118.6 (CH), 127.3 (CH), 127.7 (CH), 128.2 (CH), 128.6 (CH), 137.8 (C), 139.8 (C), 142.1 (C), 159.0 (C); IR (ATR/cm^{-1}) 1198, 1305, 1514, 1575, 3356; HRMS (ESI-TOF) calcd. for [M–H]$^-$ $C_{12}H_8NO_3$: 214.0509, found: 214.0510.

*3-(4-Chlorophenyl)-4-nitrophenol (**5d**)*

Brown solid, mp 96–97 °C. ^{1}H NMR (400 MHz, $CDCl_3$) δ 5.7–5.9 (br, 1H), 6.77 (d, *J* = 2.8 Hz, 1H), 6.88 (dd, *J* = 2.8, 8.9 Hz, 1H), 7.21 (d, *J* = 8.6 Hz, 2H), 7.38 (d, *J* = 8.6 Hz, 2H), 7.95 (d, *J* = 8.9 Hz, 1H); ^{13}C NMR (100 MHz, $CDCl_3$) δ 115.0 (CH), 118.6 (CH), 127.5 (CH), 128.7 (CH), 129.1 (CH), 134.4 (C), 136.4 (C), 138.7 (C), 141.8 (C), 159.4 (C); IR (ATR/cm^{-1}) 624, 828, 1090, 1310, 1490, 3392; HRMS (ESI-TOF) calcd. for (M^++Na^+) $C_{12}H_8ClNO_3Na$: 272.0085, found: 272.0098.

*3-(4-Trifluoromethylphenyl)-4-nitrophenol (**5e**)*

Brown solid, mp 81–82 °C. ^{1}H NMR (400 MHz, $CDCl_3$) δ 5.52–5.61 (br, 1H), 6.79 (d, *J* = 2.8 Hz, 1H), 6.93 (dd, *J* = 2.8, 9.2 Hz, 1H), 7.41 (d, *J* = 8.2 Hz, 2H), 7.68 (d, *J* = 8.2 Hz, 2H), 8.02 (d, *J* = 9.2 Hz, 1H); ^{13}C NMR (125.5MHz, $CDCl_3$): δ 115.3 (CH), 118.5 (CH), 124.0 (q, *J* = 270.8 Hz, (C)), 125.5 (q, *J* = 3.5 Hz, (CH)), 127.7 (CH), 128.2 (CH), 130.4 (q, *J* = 32.3 Hz, (C)), 138.6 (C), 141.7 (C), 141.8 (C), 159.2 (C). IR (ATR/cm^{-1}) 1126, 1168, 1325, 1503, 1575, 1614, 3395; HRMS (ESI-TOF) calcd. for (M^+) $C_{13}H_8F_3NO_3$: 283.0456, found: 283.0462.

*4-Nitro-3-(2-thienyl)phenol (**5f**)*

Brown oil. ^{1}H NMR (400 MHz, $CDCl_3$) δ 5.5–5.7 (br, 1H), 6.86 (dd, *J* = 2.7, 8.8 Hz, 1H), 6.93 (d, *J* = 2.7 Hz, 1H), 7.06–7.07 (m, 2H), 7.40 (dd, *J* = 2.1, 4.2 Hz, 1H), 7.83 (d, *J* = 8.8 Hz, 1H); ^{13}C NMR (100 MHz, $CDCl_3$) δ 115.1 (CH), 119.0 (CH), 127.0 (CH), 127.1 (CH), 127.2 (CH), 127.5 (CH), 131.8 (C), 137.4 (C), 142.7 (C), 158.5 (C); IR (ATR/cm^{-1}) 726, 906, 1340, 1517, 3433; HRMS (ESI-TOF) calcd. for (M^++Na^+) $C_{10}H_7NO_3SNa$: 244.0039, found: 244.0034.

References

[1] Fukaya, T.; Kodo, T.; Ishiyama, T.; Kakuyama, H.; Nishikawa, H.; Baba, S.; Masumoto, S. *Bioorg. Med. Chem.* **2012**, *20*, 5568–5582.

X-ray analysis

CCDC number: 2012884

All measurements were made on a Rigaku R-AXIS RAPID diffractometer using graphite monochromated Cu-Kα radiation.

The crystal-to-detector distance was 127.40 mm.

Cell constants and an orientation matrix for data collection corresponded to a primitive monoclinic cell with the dimensions:

a	=	11.3309(4) Å			
b	=	9.4834(3) Å	β	=	98.397(7)o
c	=	12.6530(4) Å			
V	=	1345.06(8) $Å^3$			

For Z = 4 and F.W. = 263.29, the calculated density is 1.300 g/cm^3. Based on the reflection conditions of:

0k0: $k = 2n$

packing considerations, a statistical analysis of intensity distribution, and the successful solution and refinement of the structure, the space group was determined to be:

*P*21 (#4)

Of the 15754 reflections were collected, where 4802 were unique (R_{int} = 0.0235); equivalent reflections were merged.

The linear absorption coefficient, μ, for Cu-Kα radiation is 7.926 cm^{-1}. An empirical absorption correction was applied which resulted in transmission factors ranging from 0.693 to 0.853. The data were corrected for Lorentz and polarization effects.

The structure was solved by direct methods [2] and expanded using Fourier techniques. The non-hydrogen atoms were refined anisotropically. Hydrogen atoms were refined using the riding model. The final cycle of full-matrix least-squares refinement [3] on F^2 was based on 4802 observed reflections and 343 variable parameters and converged (largest parameter shift was 0.00 times its esd) with unweighted and weighted agreement factors of:

$R1 = \Sigma ||F_o| - |F_c|| / \Sigma |F_o| = 0.0453$

$wR2 = [\Sigma (w (F_o^2 - F_c^2)^2)/ \Sigma w(F_o^2)^2]^{1/2} = 0.1112$

The goodness of fit [4] was 1.05. Unit weights were used. The maximum and minimum peaks on the final difference Fourier map corresponded to 0.28 and −0.15e/Å^3, respectively. The final Flack parameter [5] was 0.06(7), indicating that the present absolute structure is correct [6].

Neutral atom scattering factors were taken from International Tables for Crystallography (IT), Vol. C, Table 6.1.1.4 [7]. Anomalous dispersion effects were included in F_{calc} [8]; the values for $\Delta f'$ and $\Delta f''$ were those of Creagh and McAuley [9]. The values for the mass attenuation coefficients are those of Creagh and Hubbell [10]. All calculations were performed using the CrystalStructure [11] crystallographic software package except for refinement, which was performed using SHELXL Version 2018/3 [12].

References

[2] SIR88: Burla, M. C., Camalli, M., Cascarano, G., Giacovazzo, C., Polidori, G., Spagna, R. and Viterbo, D. (1989). J. Appl. Cryst. 22, 389-393.

[3] Least Squares function minimized: (SHELXL Version 2018/3)

$\Sigma w(F_o^2-F_c^2)^2$ where w = Least Squares weights.

[4] Goodness of fit is defined as:

$[\Sigma w(F_o^2-F_c^2)^2/(N_o-N_v)]^{1/2}$

where: N_o = number of observations

N_v = number of variables

[5] Parsons, S. and Flack, H. (2004), Acta Cryst. A60, s61.

[6] Flack, H.D. and Bernardinelli (2000), J. Appl. Cryst. 33, 114-1148.

[7] International Tables for Crystallography, Vol.C (1992). Ed. A.J.C. Wilson, Kluwer Academic Publishers, Dordrecht, Netherlands, Table 6.1.1.4, pp. 572.

[8] Ibers, J. A. & Hamilton, W. C.; Acta Crystallogr., 17, 781 (1964).

[9] Creagh, D. C. & McAuley, W.J .; "International Tables for Crystallography", Vol C, (A.J.C. Wilson, ed.), Kluwer Academic Publishers, Boston, Table 4.2.6.8, pages 219-222 (1992).

[10] Creagh, D. C. & Hubbell, J.H..; "International Tables for Crystallography", Vol C, (A.J.C. Wilson, ed.), Kluwer Academic Publishers, Boston, Table 4.2.4.3, pages 200-206 (1992).

[11] CrystalStructure 4.2.5: Crystal Structure Analysis Package, Rigaku Corporation (2000-2017). Tokyo 196-8666, Japan.

[12] SHELXL Version 2018/3: Sheldrick, G. M. (2008). Acta Cryst. A64, 112-122.

*3-Methoxy-5-(4-methylphenyl)-4-nitrocyclohexanone (**4a**)*

^{1}H NMR (400 MHz, $CDCl_3$)

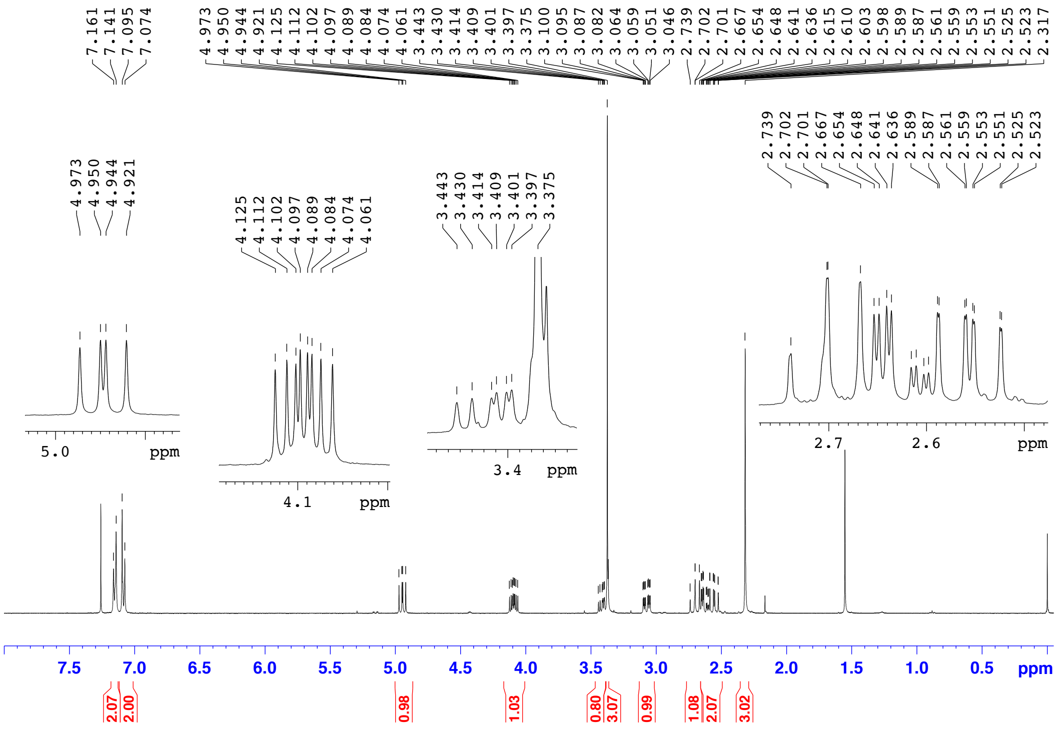

^{13}C NMR (100 MHz, $CDCl_3$)

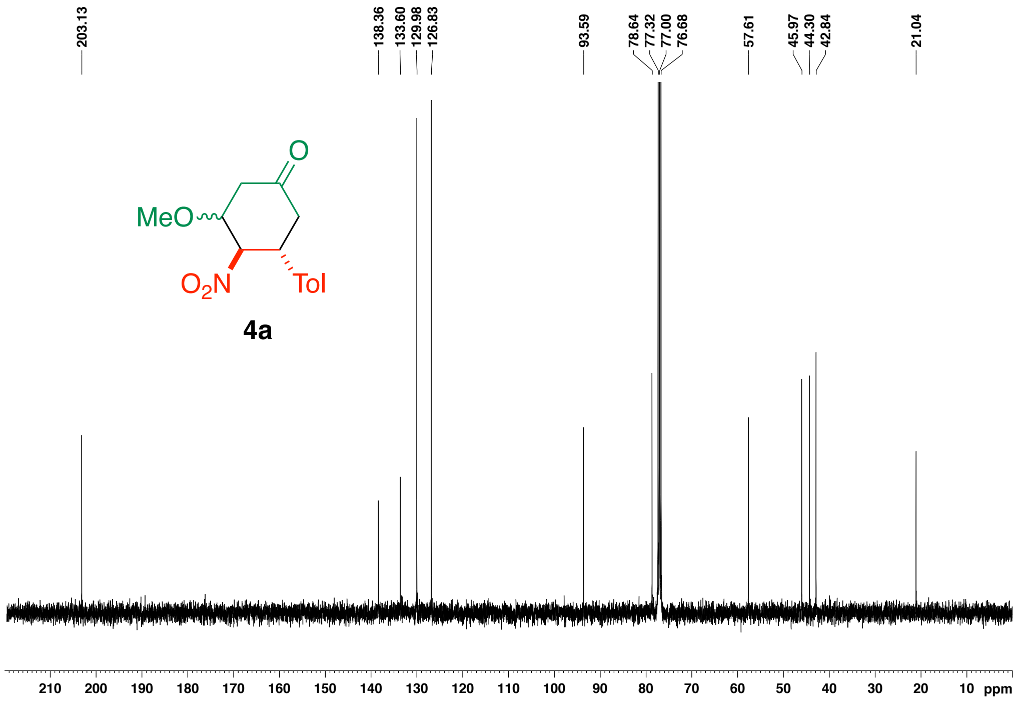

DEPT

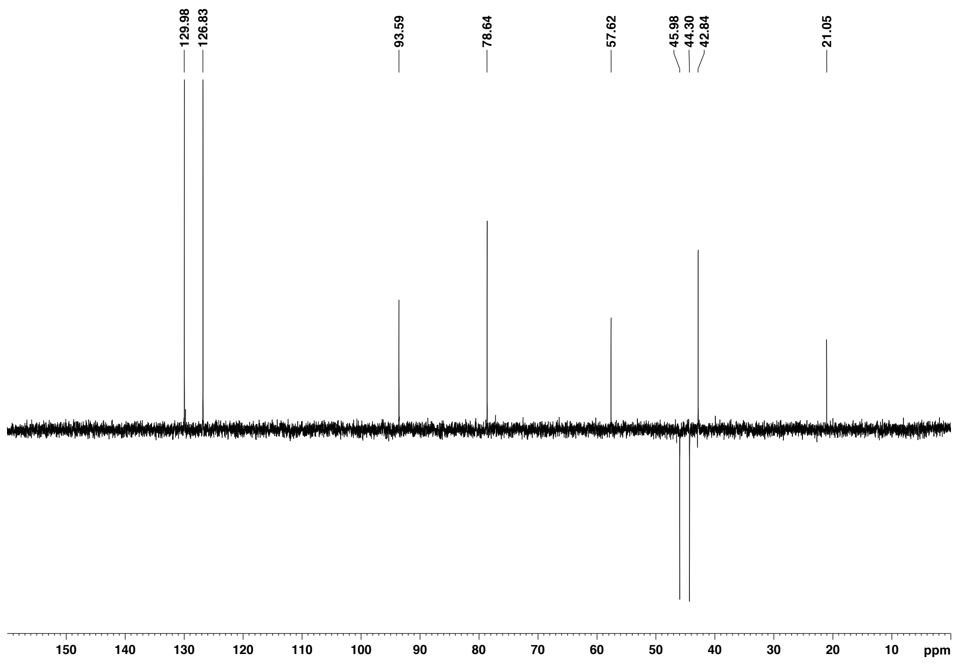

Deuteration of cyclohexanone **4a**

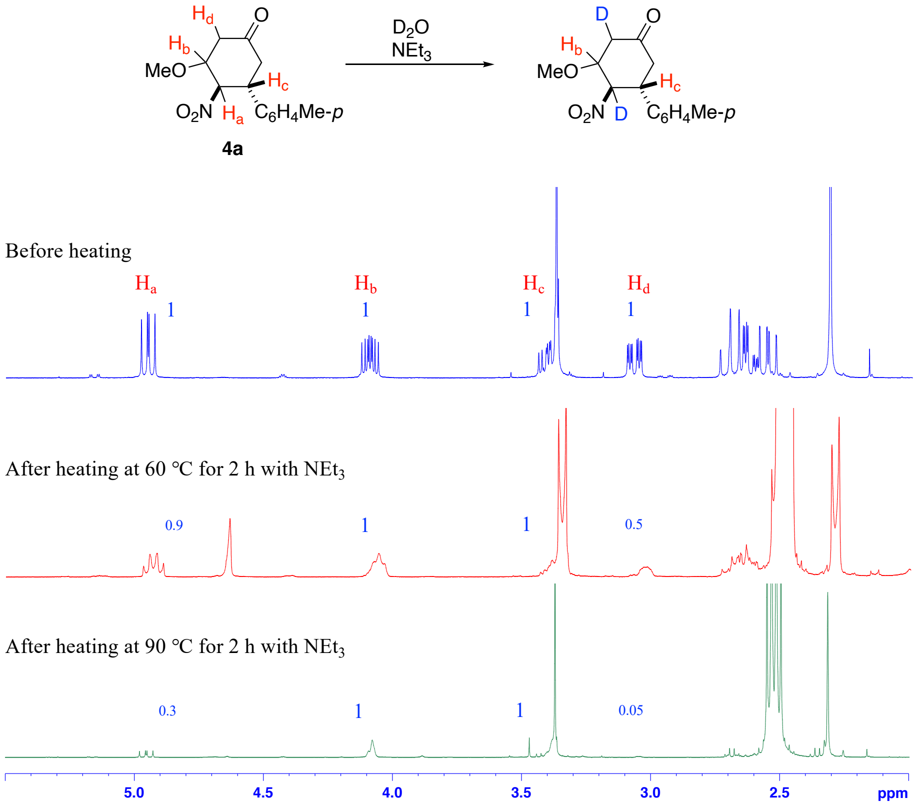

*3-(4-Methylphenyl)-4-nitrophenol (**5a**)*

^{1}H NMR (400 MHz, CDCl$_3$)

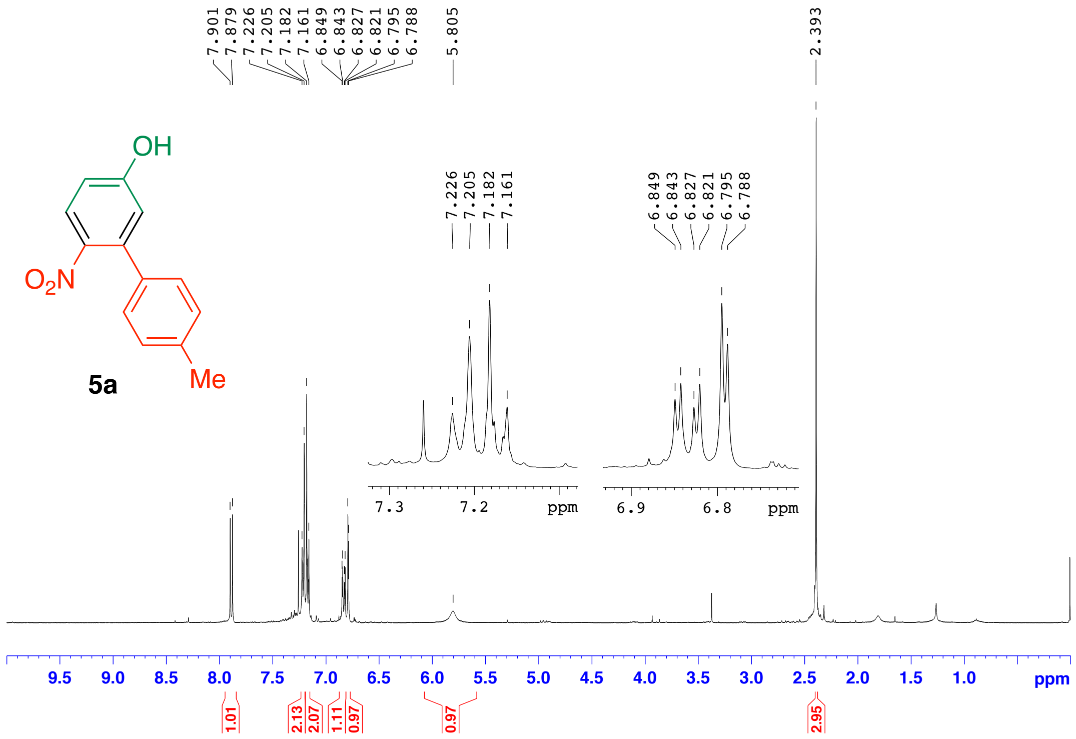

^{13}C NMR (100 MHz, CDCl$_3$)

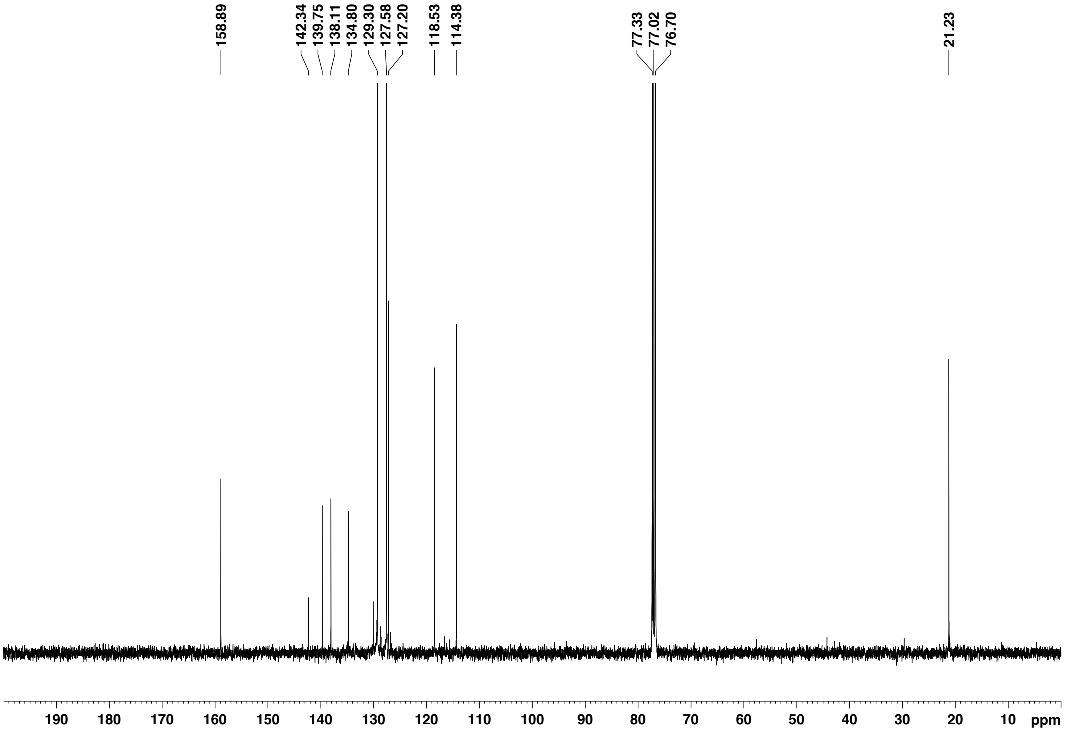

DEPT

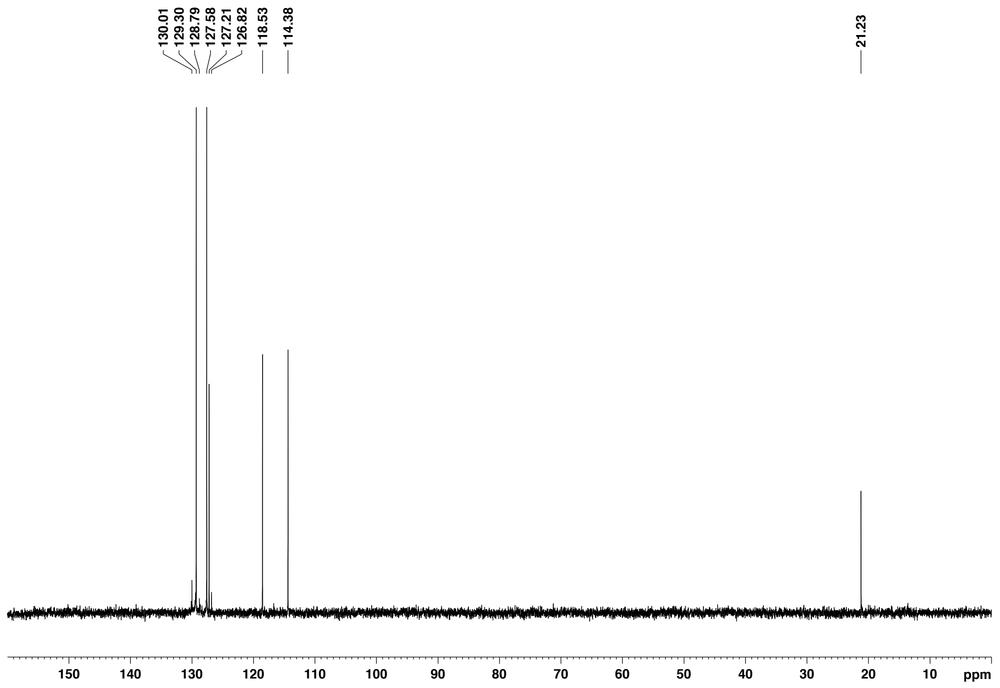
130.01
129.30
128.79
127.58
127.21
126.82
118.53
114.38
21.23
150
140
130
120
110
100
90
80
70
60
50
40
30
20
10
ppm

*3-(4-Methoxyphenyl)-4-nitrophenol (**5b**)*

^{1}H NMR (400 MHz, $CDCl_3$)

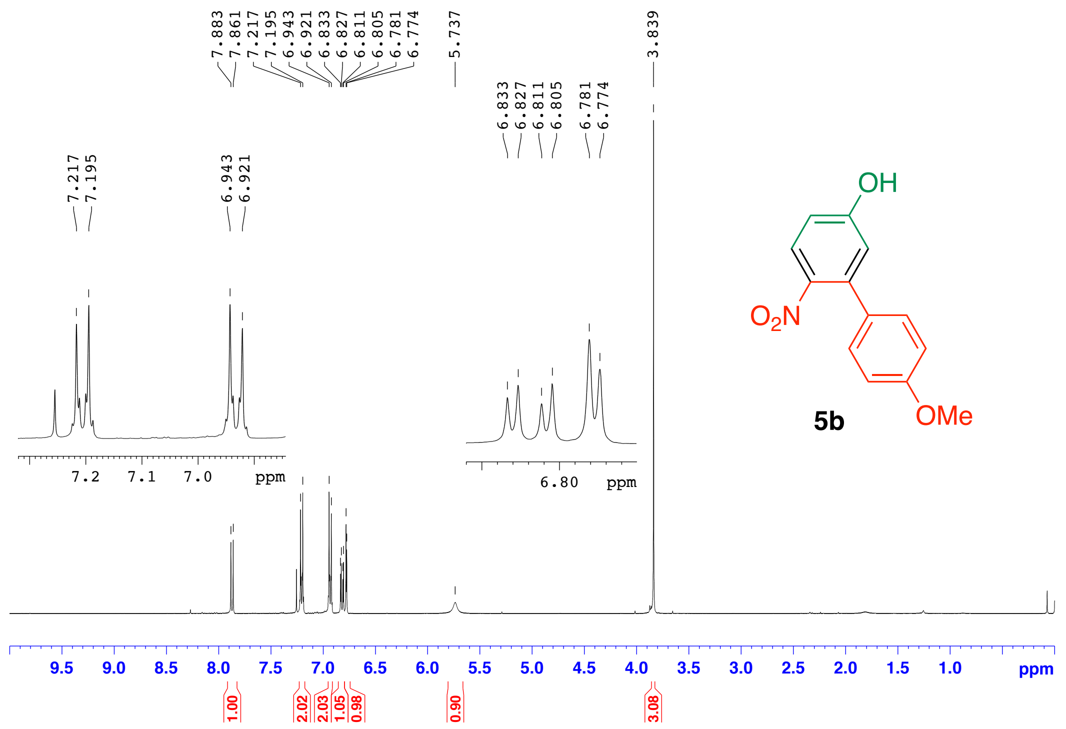

^{13}C NMR (100 MHz, $CDCl_3$)

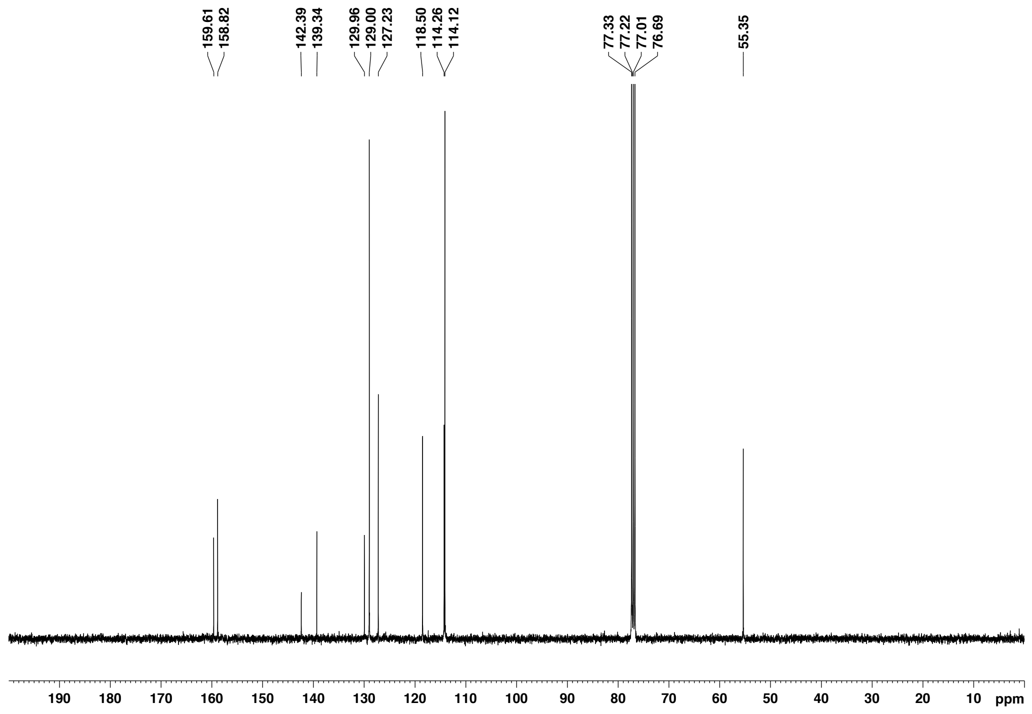

DEPT

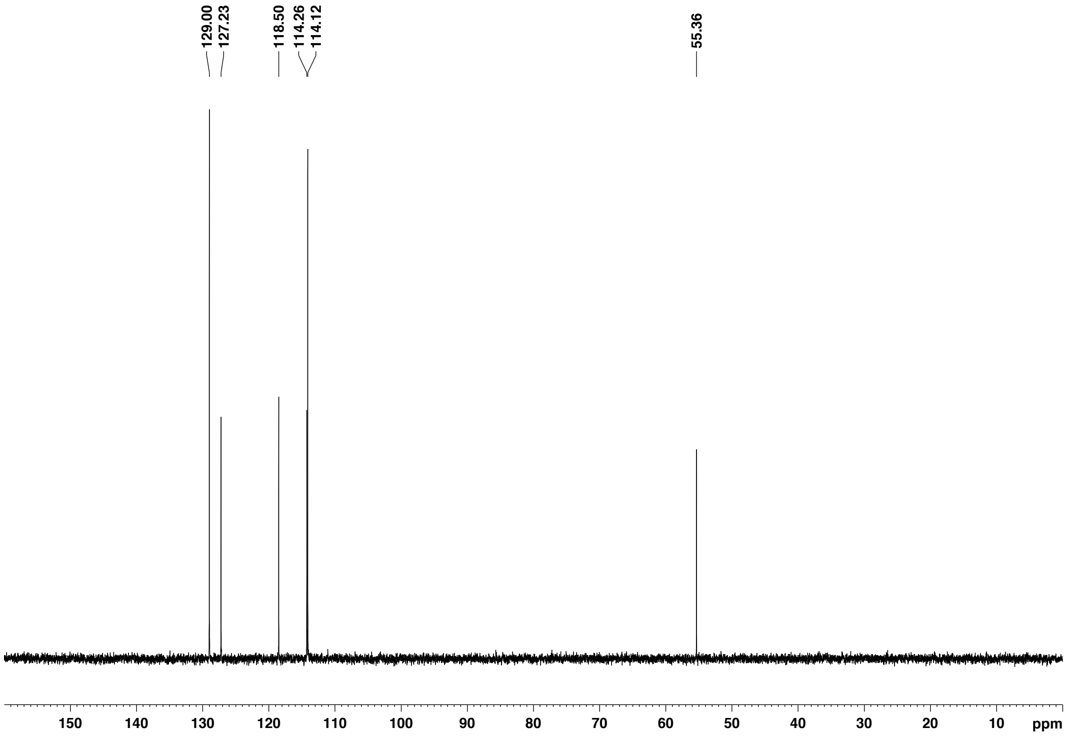

129.00
127.23
118.50
114.26
114.12
55.36
150
140
130
120
110
100
90
80
70
60
50
40
30
20
10
ppm

*4-Nitro-3-phenylphenol (**5c**)*

^{1}H NMR (500 MHz, $CDCl_3$)

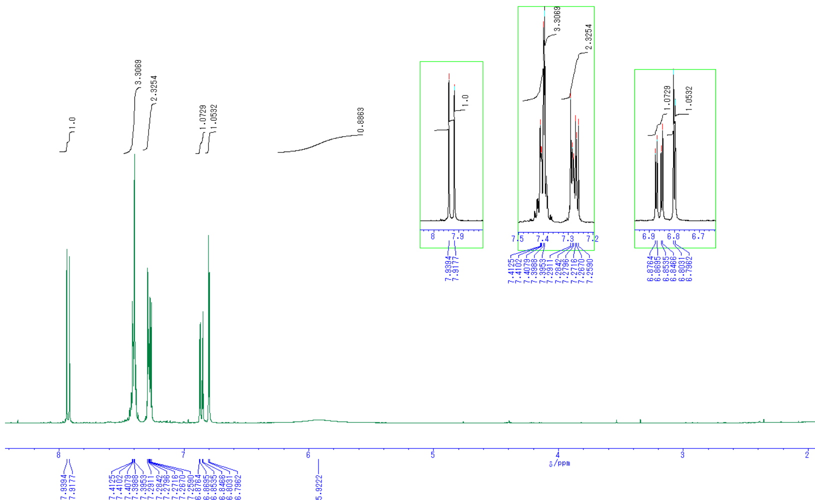

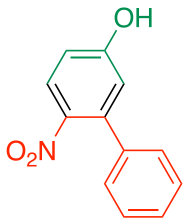

5c

^{13}C NMR (125 MHz, $CDCl_3$)

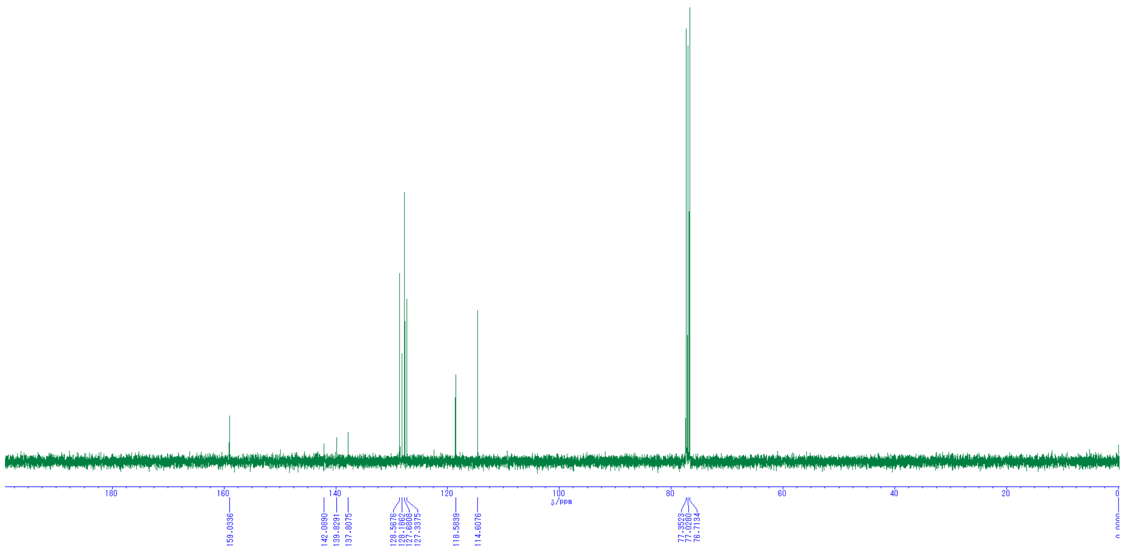

*3-(4-Chlorophenyl)-4-nitrophenol (**5d**)*

^{1}H NMR (400 MHz, $CDCl_3$)

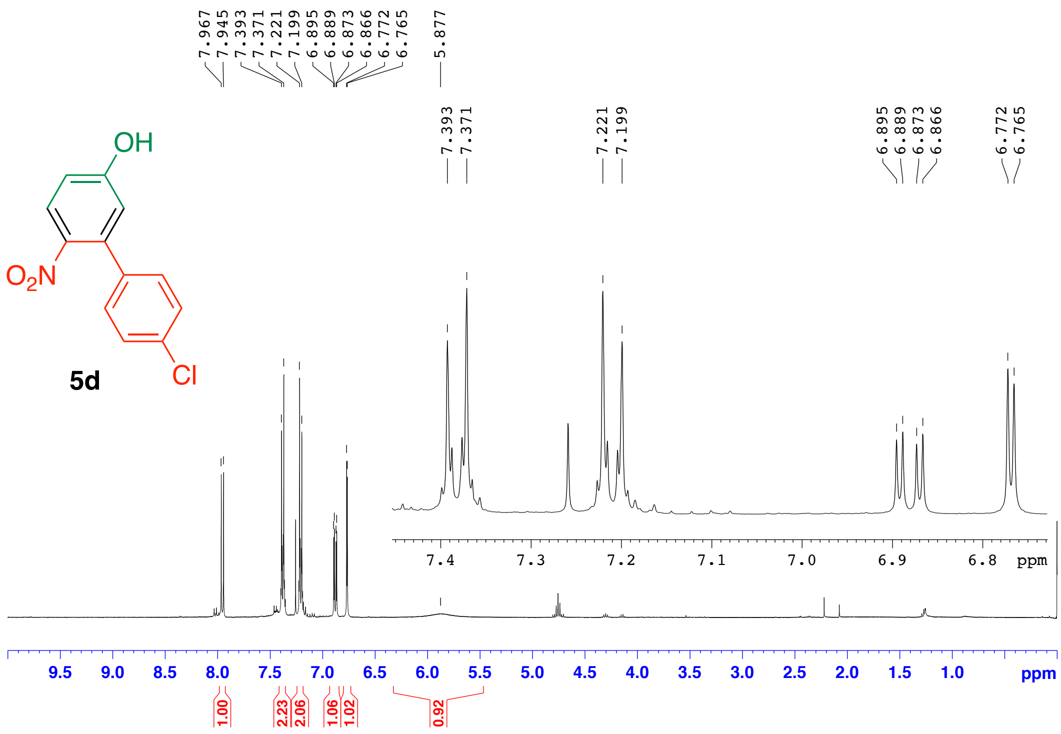

^{13}C NMR (100 MHz, $CDCl_3$)

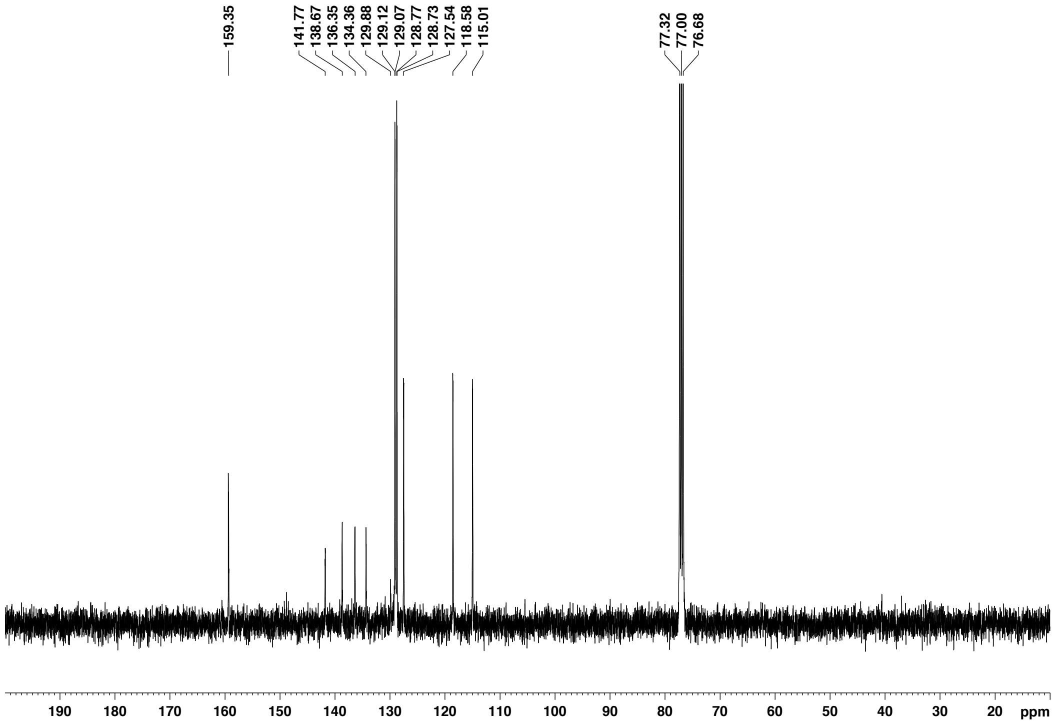

DEPT

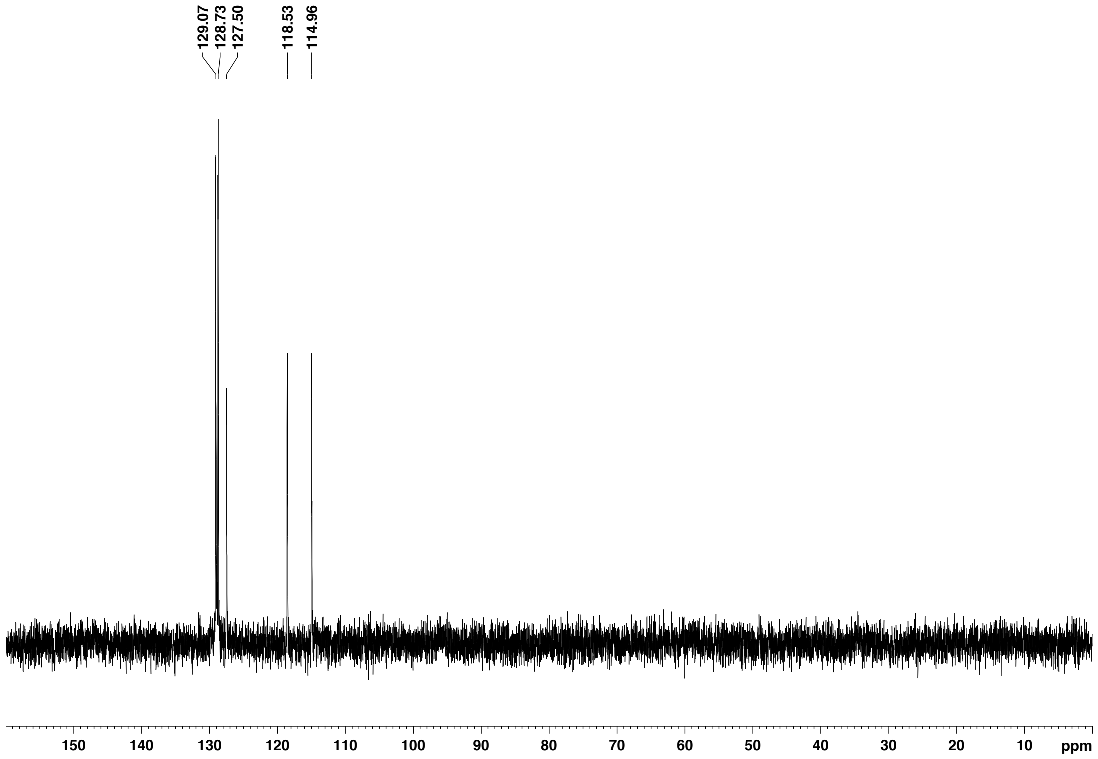
129.07
128.73
127.50
118.53
114.96
150
140
130
120
110
100
90
80
70
60
50
40
30
20
10
ppm

*3-(4-Trifluoromethylphenyl)-4-nitrophenol (**5e**)*

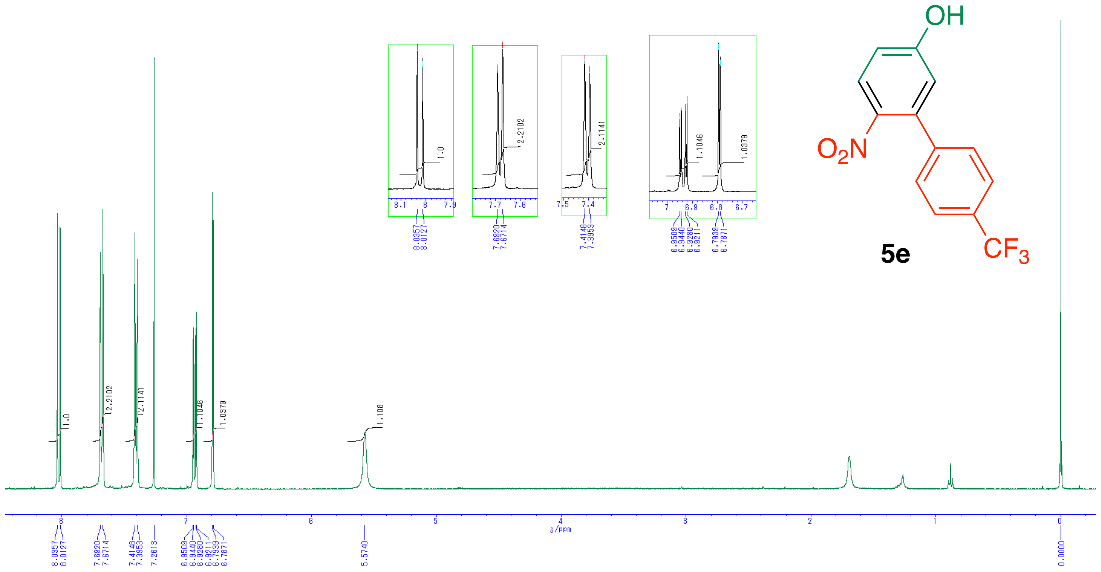

^{13}C NMR (125 MHz, $CDCl_3$)

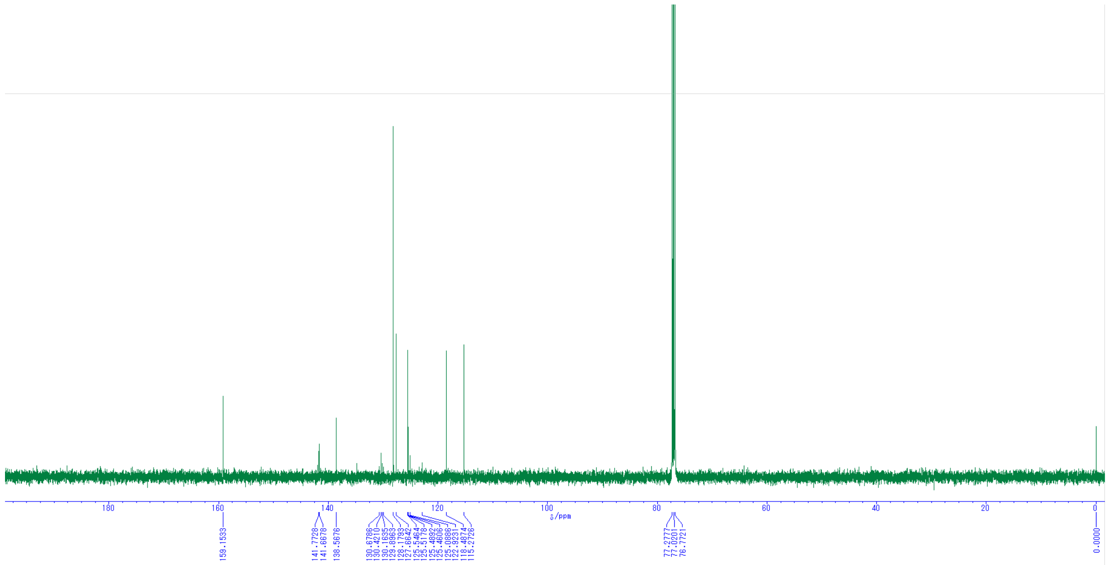

*4-Nitro3-(2-thienyl)phenol (**5f**)*

^{1}H NMR (400 MHz, $CDCl_3$)

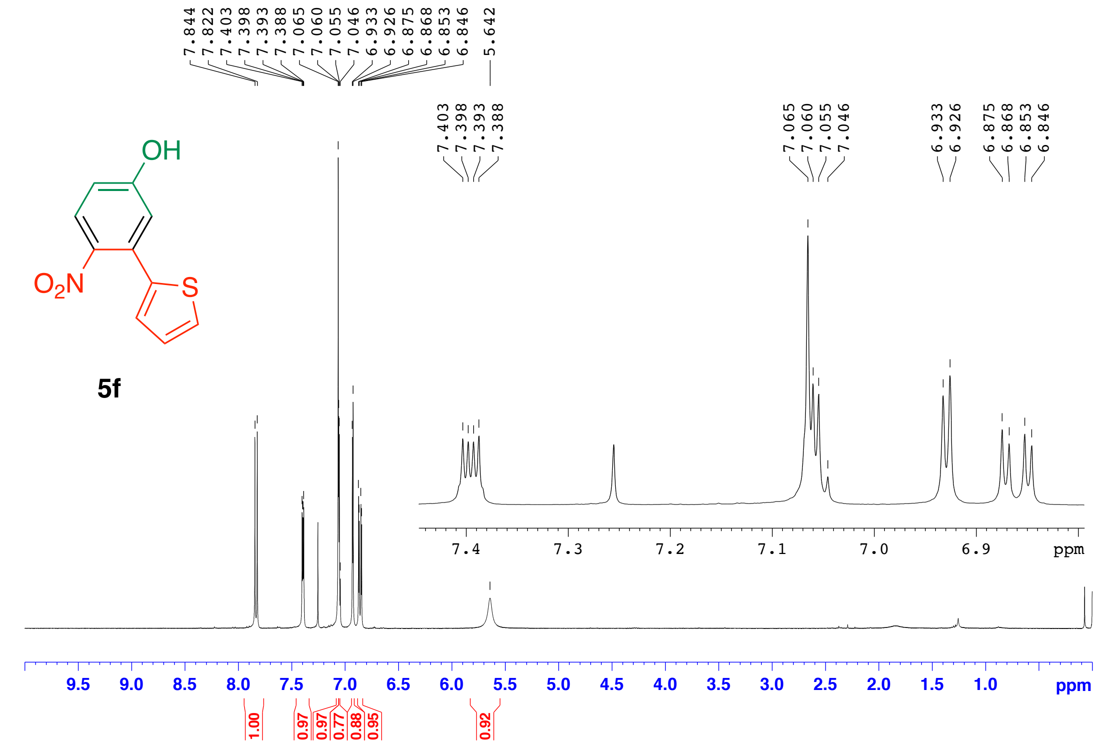

^{13}C NMR (100 MHz, $CDCl_3$)

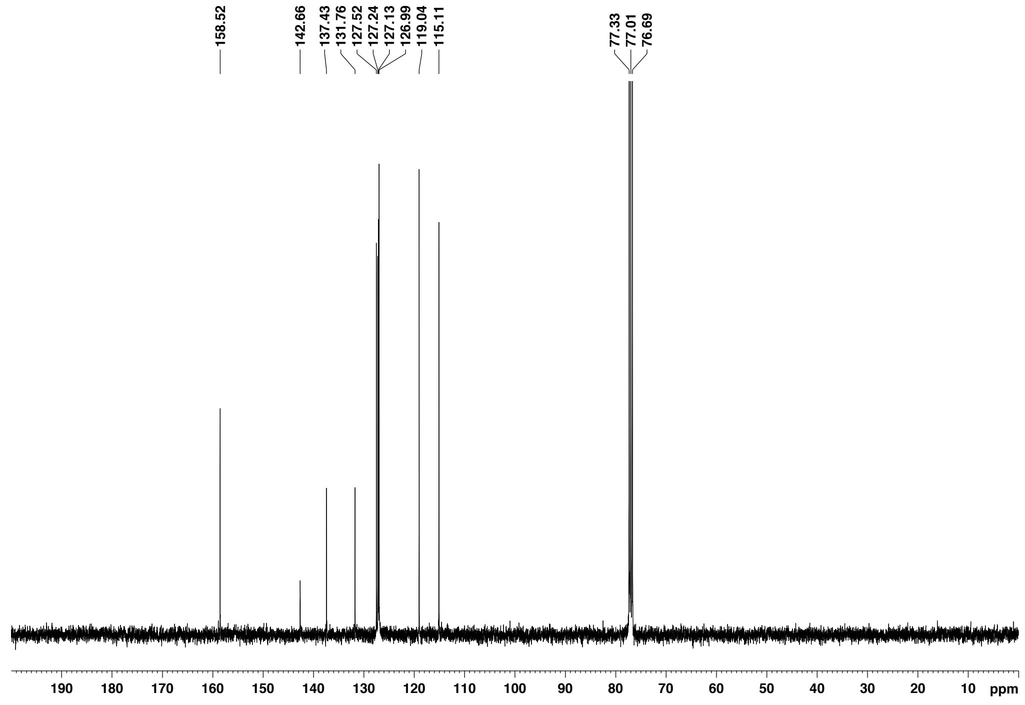

DEPT

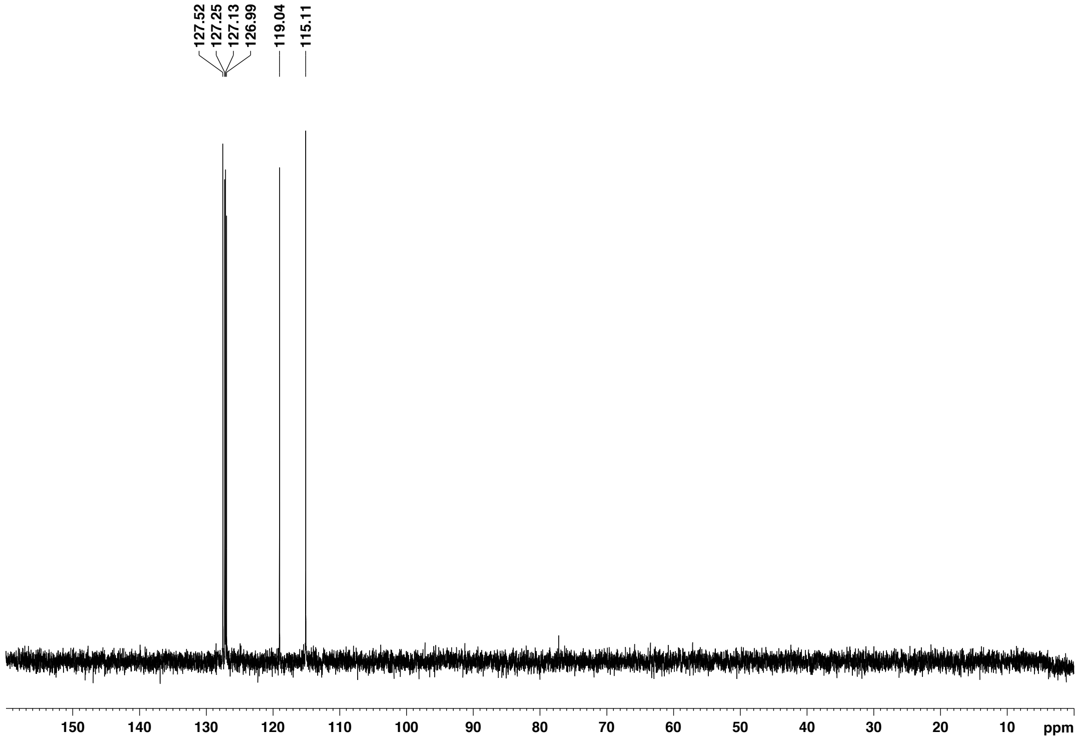
127.52
127.25
127.13
126.99
119.04
115.11
150
140
130
120
110
100
90
80
70
60
50
40
30
20
10
ppm
